# The Impact of Serum Anti-neutrophil Cytoplasmic Antibody on Clinical Characteristics and Outcomes in Pediatric-Onset Systemic Lupus Erythematosus Patients

**DOI:** 10.3389/fmed.2021.647510

**Published:** 2021-04-16

**Authors:** Chun-Chun Gau, Min-Hua Tseng, Chao-Yi Wu, Huang-Yu Yang, Jing-Long Huang

**Affiliations:** ^1^Division of Allergy, Asthma, and Rheumatology, Department of Pediatrics, Chang Gung Memorial Hospital and Chang Gung University College of Medicine, Taoyuan, Taiwan; ^2^Division of Pediatric General Medicine, Department of Pediatrics, Chang Gung Memorial Hospital and Chang Gung University College of Medicine, Taoyuan, Taiwan; ^3^Department of Pediatrics, Chang Gung Memorial Hospital at Keelung, Keelung, Taiwan; ^4^Division of Nephrology, Department of Pediatrics, Chang Gung Memorial Hospital and Chang Gung University College of Medicine, Taoyuan, Taiwan; ^5^Department of Nephrology, Chang Gung Memorial Hospital, College of Medicine, Chang Gung University, Taoyuan, Taiwan; ^6^Department of Pediatrics, New Taipei Municipal TuCheng Hospital, Chang Gung Memorial Hospital and Chang Gung University, New Taipei City, Taiwan

**Keywords:** anti-neutrophil cytoplasmic antibody, pediatric-onset systemic lupus erythematosus, lupus nephritis, hematuria, children

## Abstract

**Background:** Systemic lupus erythematosus (SLE), an autoimmune disease, is characterized by the overproduction of autoantibodies. Anti-neutrophil cytoplasmic antibodies (ANCAs) have been recognized in SLE for decades. To date, their association with SLE disease activity, especially in pediatric-onset SLE (pSLE) patients, is limited.

**Methods:** We conducted a retrospective case-control study of pSLE patients with ANCAs from 2010 to 2020. Clinical characteristics, laboratory data, renal histological features, treatment and outcomes were analyzed.

**Results:** A total of 70 pediatric-onset SLE patients (9 ANCA-positive vs. 61 ANCA-negative) with a median age of 12.23 years (age ranging from 4 years to 18 years) at diagnosis were enrolled. Among patients with ANCAs, MPO-ANCA was found in seven and PR3-ANCA in two of those cases. Patients with ANCAs had a tendency to have hematuria compared with those without ANCAs (66 vs. 24.6%, respectively; *p* = 0.026). Of the 70 SLE patients, 8 with ANCAs and 44 without ANCAs underwent renal biopsies. Patients with ANCAs (25%, 2/8) were more likely to lack the typical full-house pattern in their renal immunofluorescence (IF) staining.

**Conclusion:** pSLE patients with ANCAs tend to have hematuria and an absence of typical IF histology. However, patients with and without ANCAs showed no difference in their clinical presentations and treatment outcomes.

## Background

Systemic lupus erythematosus (SLE), a prototype of autoimmune disease with systemic involvement, is characterized by the breakdown of self-tolerance and various autoantibody production. Pediatric-onset SLE (pSLE) accounts for 15–20% of all cases and usually presents with a more aggressive clinical phenotype compared with adult-onset SLE. Although pediatric and adult lupus patients share similar clinical manifestations, lupus nephritis (LN) among the pediatric-onset population has been suggested to be different from the adult-onset form in its abrupt onset and relatively poor response to treatment (1).

Anti-neutrophil cytoplasmic antibodies (ANCAs) are a group of autoantibodies directed against cytoplasmic antigens within human neutrophils and monocytes and have been detected in patients with small vessel vasculitis and inflammatory disease (2–4). In fact, vasculitis is a common manifestation in lupus patients, and the presence of ANCAs related to clinical presentation, including interstitial lung disease, chronicity index and histopathology of lupus nephritis, has been flourishingly reported in adult lupus patients in this 5-year study (5–9). However, the association of ANCAs with pediatric-onset SLE has not been well-reviewed and has limited the discussion on the association between ANCA presence and clinical characteristics (10, 11). To date, the impact of ANCAs on renal manifestations, disease severity and clinical outcome among pediatric SLE patients remains to be elucidated.

The aim of this study was to investigate the impact of ANCAs on clinical manifestations, organ involvement and outcomes in pediatric-onset lupus patients.

## Methods

This retrospective case-control study was approved by the Ethics Committee on Human Studies at Chang Gung Memorial Hospital in Taiwan, R.O.C. (IRB 201601678A3C501). Informed consent was obtained from the patients and their parents, and all methods including chart review were performed in accordance with the relevant regulations.

### Subjects

**Figure 1:**
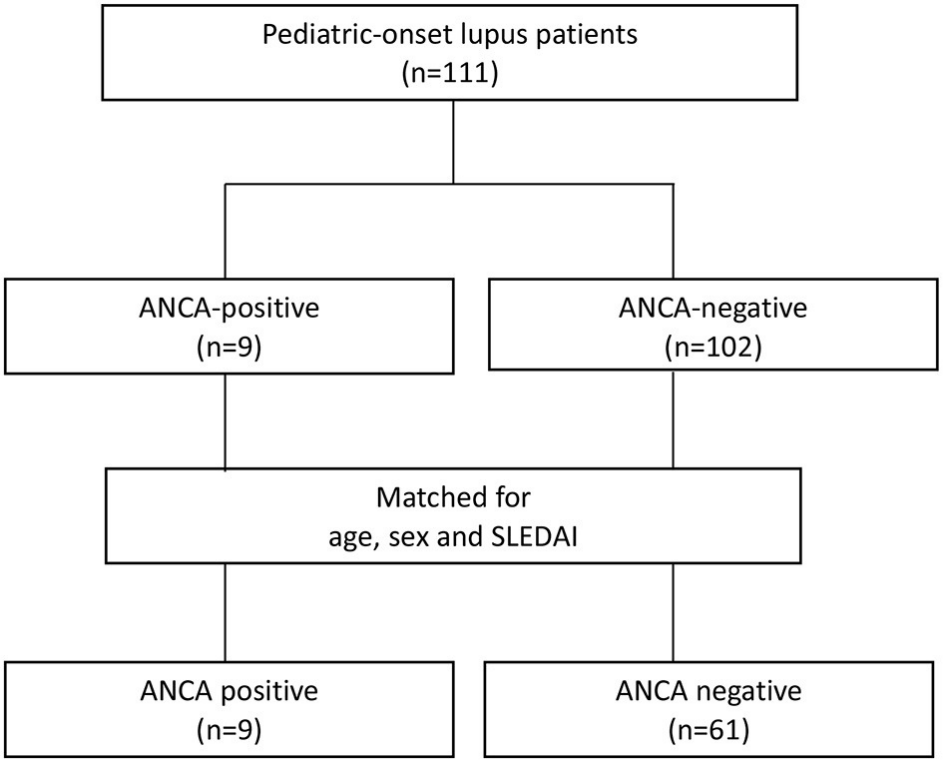
Flow chart of patient collection and categorization.

One hundred and eleven SLE patients diagnosed according to the 1997 SLE American College of Rheumatology criteria from 2010 to 2020 were recruited from a tertiary hospital (12). Patients with disease onset before the age of 18 who received regular clinical follow-up every 1–3 months for more than 6 months were enrolled (Figure 1). Nine patients with ANCAs were identified. Sixty-one patients without ANCAs were consecutively enrolled in the control group after adjustment for age, sex, and SLE disease activity index (SLEDAI) score (13).

### Clinical and Laboratory Characteristics and Outcome

The SLE-related laboratory data, including renal function (creatinine data and urinary analysis), hematology including lymphopenia (white blood count <4,000/mm^3^ more than twice), leukopenia (lymphocyte count <4,000/mm^3^ more than two times), hemolytic anemia with reticulocytosis and thrombocytopenia (platelet count <1,000.00/mm^3^ in the absence of drug use), complement C3 and C4, anti-nuclear antibody (ANA), and anti-double stranded DNA antibody (anti-dsDNA Ab), were collected, and clinical manifestations noted within the previous 10 years, including mucocutaneous presentation (malar rash, photosensitivity, discoid rash or oral ulcer), cardiovascular systems (myocarditis, pericardial effusion or pericarditis), non-erosive arthritis, pulmonary manifestations (hemoptysis or abnormality on image by radiologists) and neurological disorder (neuritis, seizure or psychosis), at presentation and follow up were recorded. Proteinuria was defined as daily protein excretion more than 4 mg/m^2^/h, 500 mg per 24 h or more than 3+ on urinalysis based on the 1997 American College of Rheumatology revised criteria (12). Hematuria was diagnosed as more than five red blood cells per high-power field. The demographic data, medications and outcomes, including acute kidney injury, chronic renal failure and all-cause mortality were also analyzed during the follow-up. Hypocomplementemia was defined as a C3 level less than the lower limit (90 mg/dl) or a C4 level less than the lower limit (10 mg/dl). Disease activity, quantified by the SLEDAI-2K score, was also calculated (13). Acute kidney injury was defined as a serum creatinine level more than 0.3 mg/ dl within 48 h or serum creatine more than 1.5 times that of the baseline value within the prior seven days (14). Furthermore, we utilized the bedside Schwartz equation to estimate the measured glomerular filtration rate (mGFR, ml/min/1.73 m^2^) status, and chronic kidney disease (CKD) was assessed at 6 months after the administration of proper treatment: stage 1, mGFR>90; stage 2, 89>mGFR>60; stage 3, 59>mGFR>30; stage 4, 29> mGFR>15 and stage 5, mGFR <15 (15, 16).

### Renal Histopathology

Fifty-two of the patients (8 positive and 44 negative for ANCAs) underwent renal biopsy due to nephritis. The histological studies, including light microscopy, immunofluorescence (IF) and electron microcopy studies, were reviewed by a pathologist who was an expert in renal diseases. The full-house pattern, which is the histological hallmark of lupus nephritis as described in the 2012 Systemic Lupus International Collaborating Clinics criteria based on their proposed classification of systemic lupus erythematosus, was defined as the presence of IgG, IgA, IgM, C1q and C3 on IF staining (12). Lupus nephritis, activity and chronicity were classified according to the International Society of Nephrology/Renal Pathology Society (17).

### Measurement of Serum ANCAs

Serum ANCA levels were routinely checked at presentation, every clinic visit and follow-up for all enrolled patients during the follow-up. Serum ANCA levels were measured by an enzyme-linked immunosorbent assay (ELISA), and an anti-MPO antibody level >5.0 U/ml and anti-PR3 antibody level >3.0 U/ml were defined as positive findings according to the manufacturer's instructions (Pharmacia Diagnostic AB, Uppsala, Sweden). In addition, defining ANCA positivity according to the ELISA method for MPO and PR3 (not as immunofluorescence assay testing positive) is an important issue, while it has been suggested that the p-ANCA pattern in the immunofluorescence test could be misinterpreted due to the interference of ANA (6, 18).

### Statistical Analyses

Data were analyzed using Student's *t* test to compare the means between two groups of continuous data. The chi-square test or Fisher's exact test (if any value was <5 in all comparisons) was used for categorical data to assess differences in clinical characteristics and evaluate the result of renal biopsy, medication and outcomes. Moreover, 95% confidence intervals were applied. Statistical analysis was performed with SPSS Statistics version 22.0.0 (SPSS Inc., Chicago, United States), and a *p*-value <0.05 was considered indicative of statistical significance.

## Results

### Demographic and Clinical Manifestations of pSLE Patients With ANCAs

As shown in Table 1, nine patients (one male and eight females) with a mean age of 12.6 ± 1.5 years (ranging from 10 to 15 years) and ANCA positivity were enrolled over 10 years. Seven and two patients had anti-MPO antibody and anti-PR3 antibody, respectively. Eight patients had proteinuria or hematuria at the initial presentation and underwent renal biopsy. Two patients had biopsy-proven membranous glomerulopathy and cast nephropathy without full-house deposition on IF staining. Seven, four, two and one patient had hematological presentations, mucocutaneous lesions, arthritis and serositis, respectively. The cases with high ANCA levels (ANCA level >100) had severe extra-renal presentations; specifically, case 4 had pulmonary hemorrhage and case 6 had vestibulocochlear neuritis. Four patients (cases 4, 5, 6, and 7) with high serum ANCA levels of more than 100 IU/ml had acute kidney injury at presentation and reached chronic kidney disease during follow up. After 6 month treatment, two patients had persistent hematuria.

**Table 1:** Clinical Data of Lupus patients with Anti-neutrophil cytoplasmic antibodies (ANCA) at the first diagnosis.

	**1**	**2**	**3**	**4**	**5**	**6**	**7**	**8**	**9**
Onset age (year)	14	11	13	13	14	15	14	10	12
Follow-up duration (months)	195	124	124	19	81	53	21	99	228
Sex	Female	Female	Female	Male	Female	Female	Female	Female	Female
**Laboratory data at presentation**									
ANA (Normal range <1:80)	-	+	+	+	+	+	+	+	+
Anti-dsDNA antibodies^*^(Normal range <130 WHO unit/mL)	83.5	35.6	490.1	49.1	195.3	47	351.6	40.5	221.3
ANCA, p-ANCA (Normal range <5 IU/m**l**)	6.1	11.8	11	134.7	134	146	146	-	-
ANCA, c-ANCA (Normal range <3 IU/m**l**)	-	-	-	-	-	-	-	5.7	3.3
Hypocomplementemia/ (Normal range C3 >90 or C4>10 mg/d**l**)	+	+	+	+	-	+	+	+	+
Urine protein	Negative	18.2mg/m^2^/h	12.8 mg/m^2^/h	79.8 mg/m^2^/h	30mg/dl	77.6 mg/m^2^/h	718 24 mg/m^2^/h	118.7 mg/m^2^/h	1+
Hematuria	-	+	+	+	+	-	+	+	-
Serum albumin (Normal range 2.8–5.4 g/d**l**)	-	4.6	2.86	3.09	3.79	3.86	2.9	3.25	4.8
Serum creatinine^**^ (Normal range 0.2–1.0 mg/dl)	-	0.47	0.34	0.91	0.65	0.49	5.39	0.36	0.51
Renal pathology									
Light microscopy	-	LN III	Cast nephropathy	LN IV	LN V	LN V	LN V	LN IV	LN IV
Full house	-	+	(IgG 1+)	+	(IgG 3+)	+	+	+	+
**Organ involvement at presentation**									
Kidney	-	+	+	+	+	+	+	+	+
Hematology	+	-	+	+	+	-	+	+	+
Dermatology	-	+	-	+	-	-	-	+	+
Joints	+	+	-	-	-	-	-	-	-
Heart	-	-	+	-	-	-	-	-	-
Neurology	-	-	-	-	-	+	-	-	-
Pulmonary	-	-	-	+	-	-	-	-	-
**Treatment**									
Steroid	+	+	+	+	+	+	+	+	+
Azathioprine	+	-	+	-	-	+	-	_	+
HCQ	+	+	+	-	-	-	-	+	+
MMF	-	+	-	+	+	+	+	-	-
Others	-	Cyclosporine,	-	Plasma	Cyclosporine	-	Plasma	Cyclophosphamide	-
		Cyclophosphamide		exchange			exchange		
**Outcome**									
Persistent hematuria	-	-	-	+	+	-	-		-
Chronic kidney disease (stage)	-	-	-	2	1	1	3	1	-

LN, lupus nephritis; HCQ, hydroxychloroquine; MMF, mycophenolic acid.

* Anti-double stranded DNA (dsDNA) antibodies (WHO unit/mL),

** serum creatinine (mg/dl) at biopsy.

*+Case 1 did not have the indication for renal biopsy*.

### Comparison of pSLE Patients With and Without ANCAs

#### Demographic Data

Sixty-one pSLE patients were enrolled in the control group. As shown in Table 2, there were no differences between patients with and without ANCAs with respect to sex (88.9 vs. 90.2% were female), age at diagnosis (12.93 ± 3.28 vs. 13.02 ± 1.63 years old) or follow-up duration (98.73 ± 68.43 vs. 132.16 ± 73.14 months).

**Table 2:** Clinical characteristics of pediatric-onset SLE patients with and without anti-neutrophil cytoplasmic antibodies (ANCAs).

	**ANCA-positive** **(*n* = 9)**	**ANCA-negative** **(*n* = 61)**	***P*-value**
Female (%)	8 (88.9%)	55 (90.2%)	0.905
Onset age (year)	12.93 ± 3.28	13.02 ± 1.63	0.937
Follow-up (months)	98.73 ± 68.43	132.16 ± 73.14	0.368
SLEDAI^+^ at presentation	12.11 ± 6.92	12.95 ± 7.11	0.314
**Laboratory data at presentation**			
C3 (g/L)	66.49 ± 29.67	54.31 ± 35.51	0.334
C4 (g/L)	11.86 ± 7.32	9.75 ± 7.54	0.449
Anti-dsDNA antibodies	178.68 ± 156.60	240.76 ± 150.78	0.948
(unit/mL)			
Creatinine (mg/dL)	1.16 ± 1.62	0.68 ± 0.43	0.059
WBC (10^6^/L)	6255.56 ± 4143.40	5417.54 ± 2930.88	0.455
Platelets (10^6^/L)	189.78 ± 93.03	180.51 ± 113.32	0.817
Hemoglobin (g/L)	9.86 ± 2.75	11.30 ± 2.91	0.168
Proteinuria (%)	4 (50%)	41 (67.2%)	0.435
Hematuria (%)	6 (66.6%)	15 (24.6%)	0.026^*^
**Renal histology**			
Activity	8.25 ± 2.63	7.28 ± 4.39	0.64
Chronicity	0.5 ± 1.00	2.30 ± 2.48	0.11
**Treatment at presentation and during follow-up, numbers**			
Prednisolone (%)	9 (100%)	61(100%)	N/A
Azathioprine (%)	5 (55.6%)	44 (72.1%)	0.128
Cyclophosphamide (%)	3 (33.3%)	23 (37.7%)	1.000
Mycophenolic acid (%)	3 (33.3%)	31 (50.8%)	0.479
Hydroxychloroquine (%)	6 (66.7%)	43 (70.5%)	1.000
Cyclosporine (%)	1 (11.1%)	12 (19.7%)	1.000
Rituximab (%)	0 (0%)	3 (4.9%)	1.000
**Outcome, numbers**			
Acute kidney injury (%)	1 (11.1%)	3 (4.9%)	0.538
Chronic renal disease (%)	5 (55.6%)	29 (47.5%)	0.970
End-stage renal disease (%)	0 (0%)	4 (6.6%)	0.143
Death (%)	0 (0%)	1 (1.7%)^**^	0.696

+ SLEDAI, SLE disease activity index;

** Died of sepsis,

* *p < 0.05*.

#### Laboratory Characteristics

There were no significant differences in serological characteristics, including complement, anti-dsDNA antibody, serum creatinine, white blood cell count, platelet count and hemoglobin, between patients with and without ANCAs at the time of diagnosis. However, patients with ANCAs had a higher rate of initial hematuria than those without ANCAs (62.5 vs. 24.6%, respectively; *p* = 0.026), as shown in Table 2.

#### Organs Involved

**Figure 2:**
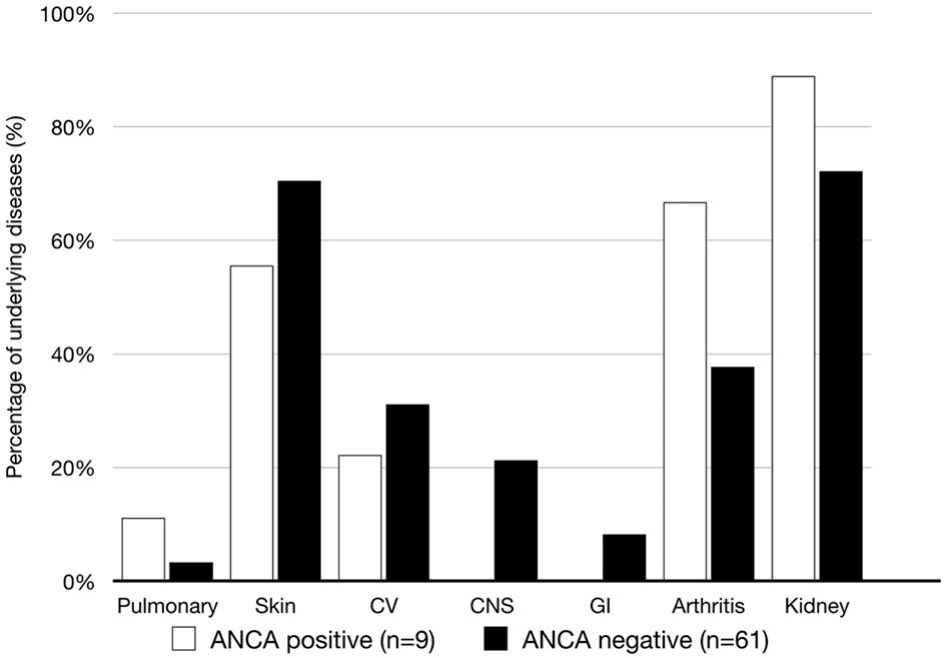
Organ systems involved in the patients with and w ithout ANCAs during the follow-up were investigated. CV, cardiovascular; GI, gastrointestinal.

As shown in Figure 2, skin, cardiovascular and joint involvements were the most common manifestations in patient with ANCA. Only patients without ANCAs had gastrointestinal organ damage. However, there was no significant difference in organ involvement between the two groups.

#### Renal Histology

**Figure 3:**
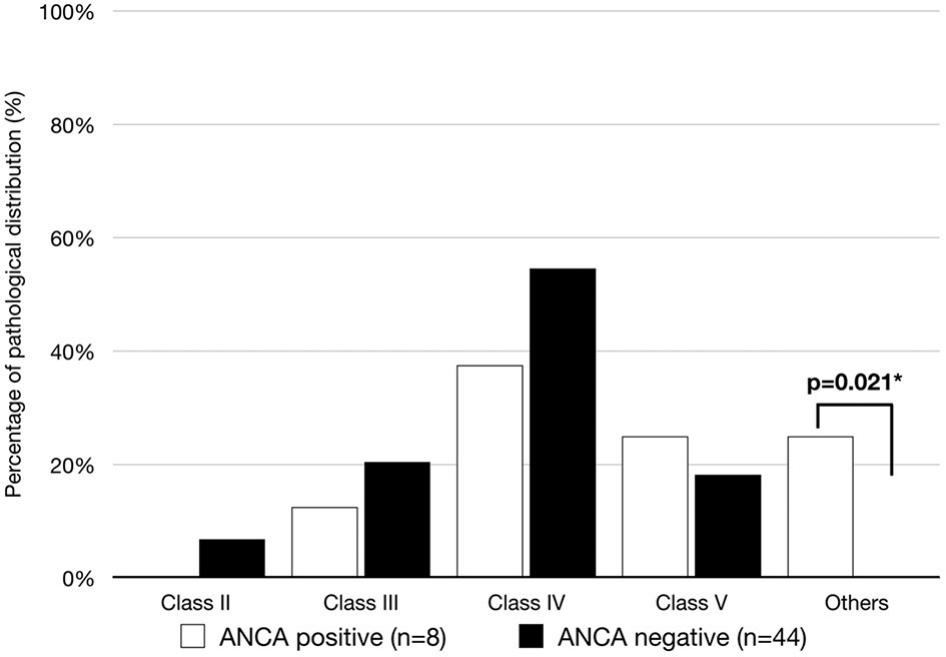
Patients with biopsy with ISN classes of lupus nephritis in the ANCA-positive and ANCA-negative groups. A significant difference was observed in the ANCA-positive group, but the classic full-house deposition was absent (*p*-value <0.05).

The lack of typical full-house IF staining was revealed in two patients with ANCAs but not in patients without ANCAs (25 vs. 0%, *p* = 0.021). In patients with ANCAs, the histological features were class IV glomerulonephritis (3, 37.5%), class V glomerulonephritis (2, 25%), class I glomerulonephritis (1, 12.5%) and nephritis with an absence of glomerulonephritis (2, 25%). As shown in Figure 3, in the 44 lupus patients without ANCAs, class IV was also the most prevalent histopathology (24, 54.5%), followed by class II, class III and class IV glomerulonephritis (3, 6.8%; 9, 20.4%; 9,18.2%; respectively).

#### Treatment

As shown in Table 2, all of the patients received steroid treatment. Immunosuppressants, including azathioprine and hydroxychloroquine, were used if there was hematological or mucocutaneous involvement. For the patients with biopsy-proven proliferative lupus nephritis, intravenous cyclophosphamide or mycophenolic acid (MMF) was administered. In addition, rituximab and cyclosporin were administered in cases of poor response to each of these drugs. There were no obvious differences between patients with or without ANCAs.

#### Outcomes

During the observation period, in the ANCA group, one patient had acute kidney injury and five had chronic kidney disease after 6 months of treatment, while three patients had acute kidney injury and twenty-nine had chronic kidney disease in the non-ANCA group, which did not correspond to a significant difference. Of the patients without ANCAs, four needed hemodialysis, and one died due to sepsis during follow up. In contrast to the patients without ANCAs, none of the nine patients with ANCAs progressed to renal failure or death during the follow-up period. However, no significant difference in mortality between patients with and without ANCAs was found.

## Discussion

This was the first longitudinal study that analyzed the impact of ANCAs on pSLE patients clinical implications. Our results indicate that the positive ANCA titers in pSLE cases are significantly associated with hematuria. In cases with positive ANCAs, two distinct histopathologies and atypical presentation without the full-house pattern of deposition in IF staining were discovered. Furthermore, in the case analysis, obvious renal manifestation as hematuria was noted in positive ANCA cases, and some patients with a high ANCA level also had pulmonary hemorrhage and neurological manifestations. Therefore, we focused on determining the renal association of hematuria and IF staining.

During the Hill et al. clinical follow-up, patients with ANCA-associated vasculitis had persistent hematuria (17). In addition, Mahoney et al. noted that persistent hematuria may be observed in ANCA vasculitis in adults, but it is not related to recurrence (19). In adult lupus patients with ANCAs, however, Pyo et al. reported no increased incidence of hematuria (5). To date, only two studies have mentioned the clinical presentation in pSLE with ANCAs, but neither studied the clinical manifestations of hematuria (10, 20). As indicated by Wang et al., the ANCA was thought to relate to neutrophil extracellular trap (NET) formation, which caused cell death and provided a novel source of autoantigens that resulted in poor renal outcome of lupus patients (21). Additionally, in our study, we found that pediatric-onset SLE patients may be prone to renal damage, which is considered to be related to ANCAs with hematuria.

The typical renal histopathological presentation of patients with ANCA-associated systemic vasculitis includes necrotizing vasculitis. According to previous reports, crescentic glomerulonephritis and global sclerosing were the most common renal histopathologies among patients with ANCA-associated renal vasculitis (22). In addition, Turner-Stokes et al. found a high frequency of lupus class IV-S lupus nephritis in ANCA-positive adult patients (8). Furthermore, recent studies confirmed that ANCA-associated vasculitis is related to the chronicity index in adults (5). However, there was no significant difference in treatment outcome or disease severity (8). After reviewing these 8 cases with ANCA-associated LN, we did not find evidence of differences in outcomes, treatment, crescent formation, classification or chronicity index in pSLE patients in the presence or absence of ANCAs. However, while examining the deposition of immunofluorescence, the renal biopsy samples lacked the full-house pattern present in the two pSLE patients with ANCAs. Although the relationship between the mechanism of ANCA vasculitis and lupus needs further investigation, the early recognition of ANCAs in pSLE patients may result in favorable and appropriate outcomes in contrast to the results of a previous study. However, this may contribute to a faster and more intensive treatment for patients with than without ANCAs.

In a review of laboratory data, Su et al. found that ANCA-positive patients with new-onset adult SLE may have higher serum creatinine levels than ANCA-negative patients (9). However, the laboratory data or renal histopathological presentation were never reviewed in pSLE patients with ANCA. In our study, although not statistically significant, the serum creatinine of patients with ANCAs was higher than that of patients without ANCAs in our study. However, the lack of significance may be due to the small number of subjects in this case or the quicker intervention than that received by the adult lupus nephritis patients.

Furthermore, as is already known, a positive ANCA titer may be related to interstitial lung disease (7). Yu et al. conducted a study on lupus patients with ANCAs that indicated no obvious difference in mucocutaneous, gastrointestinal and arthritis aspects but indicated a propensity for lupus myocarditis (23). An evidence study indicated that lupus patients with ANCAs are more likely to have severe neurological presentations, including psychosomatic manifestations (24). In a Faure-Fontenla study, pSLE patients with positive ANCA did not have evidence of vasculitis-associated features or specific organ involvement (10). This is also in accordance with research reporting no obvious extra-renal characteristics.

These were some limitations of our study. One is the small population due to the low prevalence [16% prevalence of ANCA in patients with lupus nephritis (21)] in pSLE patients, which may mask the possibility of complications, and we had to select appropriate control cases with the same disease severity for analysis. Furthermore, although the treatment may have selection bias, we treated our patients according to the current standard protocol (25).

## Conclusion

Currently, hematuria and the lack of a full house were found in pediatric lupus patients with ANCA, and patients with or without ANCAs showed no difference in their clinical presentations and treatment outcomes. Further study is warranted to verify our findings.

## Data Availability Statement

The original contributions presented in the study are included in the article/supplementary material, further inquiries can be directed to the corresponding author/s.

## Ethics Statement

The studies involving human participants were reviewed and approved by This retrospective case-control study was approved by the Ethics Committee on Human Studies at Chang Gung Memorial Hospital in Taiwan, R.O.C. (IRB 201601678A3C501). Informed consent was obtained from the patients and their parents. Written informed consent to participate in this study was provided by the participants' legal guardian/next of kin.

## Author Contributions

C-CG, M-HT, C-YW, H-YY, and J-LH: study conception and design. C-CG: acquisition of data and drafting the article. C-CG, M-HT, C-YW, and H-YY: analysis and interpretation of data. M-HT and C-YW: revising the article. All authors have finally approved the submitted version to be published.

## Conflict of Interest

The authors declare that the research was conducted in the absence of any commercial or financial relationships that could be construed as a potential conflict of interest.

## References

[1] Aggarwal A Srivastava P. Childhood onset systemic lupus erythematosus: how is it different from adult SLE? Int J Rheum Dis. (2015) 18:182–91. 10.1111/1756-185x.12419 24965742

[2] Kirito Y Yamamoto D Uchiyama T. Proteinase 3-antineutrophil cytoplasmic antibody-positive ulcerative colitis presenting with abducens neuropathy. BMJ Case Rep. (2017) 2017:bcr2016218353. 10.1136/bcr-2016-218353 28069788 PMC5256449

[3] Mahler M Damoiseaux J Ballet V Dillaerts D Bentow C Cohen Tervaert JW. PR3-anti-neutrophil cytoplasmic antibodies (ANCA) in ulcerative colitis. Clin Chem Lab Med. (2017) 56:e27–e30. 10.1515/cclm-2017-0346 28755529

[4] Olbjorn C Cvancarova Smastuen M Thiis-Evensen E Nakstad B Vatn MH Perminow G. Serological markers in diagnosis of pediatric inflammatory bowel disease and as predictors for early tumor necrosis factor blocker therapy. Scand J Gastroenterol. (2017) 52:414–9. 10.1080/00365521.2016.1259653 27887202

[5] Pyo JY Jung SM Song JJ Park YB Lee SW. ANCA positivity at the time of renal biopsy is associated with chronicity index of lupus nephritis. Rheumatol Int. (2019) 39:879–84. 10.1007/s00296-019-04263-2 30806732

[6] Weinstein A. ANCA in lupus nephritis. Lupus. (2018) 27:522. 10.1177/0961203317734925 29058992

[7] Alba MA Flores-Suarez LF Henderson AG Xiao H Hu P Nachman PH. Interstital lung disease in ANCA vasculitis. Autoimmun Rev. (2017) 16:722–9. 10.1016/j.autrev.2017.05.008 28479484 PMC6343660

[8] Turner-Stokes T Wilson HR Morreale M Nunes A Cairns T Cook HT. Positive antineutrophil cytoplasmic antibody serology in patients with lupus nephritis is associated with distinct histopathologic features on renal biopsy. Kidney Int. (2017) 92:1223–31. 10.1016/j.kint.2017.04.029 28750930 PMC5652376

[9] Su F Xiao W Yang P Chen Q Sun X Li T. Anti-neutrophil cytoplasmic antibodies in new-onset systemic lupus erythematosus. An Bras Dermatol. (2017) 92:466–9. 10.1590/abd1806-4841.20175476 28954092 PMC5595590

[10] Faure-Fontenla MA Rodriguez-Suarez RS Arias-Velasquez R Garcia-Gonzalez JE. Antineutrophil cytoplasmic antibodies in systemic lupus erythematosus in childhood. J Rheumatol. (1999) 26:2480–1. 10555913

[11] Bakkaloglu A Topaloglu R Saatci U Ozdemir S Ozen S Bassoy Y. Antineutrophil cytoplasmic antibodies in childhood systemic lupus erythematosus. Clin Rheumatol. (1998) 17:265–7. 9694070 10.1007/BF01451065

[12] Petri M Orbai AM Alarcón GS Gordon C Merrill JT Fortin PR. Derivation and validation of the Systemic Lupus International Collaborating Clinics classification criteria for systemic lupus erythematosus. Arthritis Rheum. (2012) 64:2677-86. 10.1002/art.34473 22553077 PMC3409311

[13] Bombardier C Gladman DD Urowitz MB Caron D Chang CH. Derivation of the SLEDAI. A disease activity index for lupus patients. The Committee on Prognosis Studies in SLE. Arthritis Rheum. (1992) 35:630–40. 1599520 10.1002/art.1780350606

[14] Khwaja A. KDIGO clinical practice guidelines for acute kidney injury. Nephron Clin Pract. (2012) 120:c179–84. 10.1159/000339789 22890468

[15] Schwartz GJ Haycock GB Edelmann CM Jr Spitzer A. A simple estimate of glomerular filtration rate in children derived from body length and plasma creatinine. Pediatrics. (1976) 58:259–63. 951142

[16] K/DOQI clinical practice guidelines for chronic kidney disease: evaluation classification and stratification. Am J Kidney Dis. (2002) 39(2 Suppl 1):S1–266. 11904577

[17] Hill GS Delahousse M Nochy D Tomkiewicz E Remy P Mignon F. A new morphologic index for the evaluation of renal biopsies in lupus nephritis. Kidney Int. (2000) 58:1160–73. 10.1046/j.1523-1755.2000.00272.x 10972679

[18] Sobral S Ramassur K Apsley E Isenberg D. Do anti-neutrophil cytoplasmic antibodies play a role in systemic lupus erythematosus (SLE) patients? Analysis of the University College Hospital SLE cohort. Lupus. (2018) 27:343–4. 10.1177/0961203317724218 28767004

[19] Mahoney SL Nachman PH. Persistent hematuria in ANCA vasculitis: ominous or innocuous? Clin J Am Soc Nephrol. (2018) 13:201–2. 10.2215/cjn.14101217 29371338 PMC5967444

[20] Bobek D Vukovic J Malenica B Bojanic K Rukavina I Jelusic M. Anti-neutrophil cytoplasmic antibody positivity in five children with systemic lupus erythematosus–what is the importance of this finding? Acta Dermatovenerol Croat. (2014) 22:264–70. 25580781

[21] Wang Y Huang X Cai J Xie L Wang W Tang S. Clinicopathologic characteristics and outcomes of lupus nephritis with antineutrophil cytoplasmic antibody: a retrospective study. Medicine. (2016) 95:e2580. 10.1097/MD.0000000000002580 26825903 PMC5291573

[22] Cordova-Sanchez BM Mejia-Vilet JM Morales-Buenrostro LE Loyola-Rodriguez G Uribe-Uribe NO Correa-Rotter R. Clinical presentation and outcome prediction of clinical, serological, and histopathological classification schemes in ANCA-associated vasculitis with renal involvement. Clin Rheumatol. (2016) 35:1805–16. 10.1007/s10067-016-3195-z 26852317

[23] Yu YW Liu ZR Xie D Chen SX Li HY. [Clinical significance of antineutrophil cytoplasmic antibodies in patients with lupus nephritis]. Nan fang yi ke da xue xue bao = J South Med Univ. (2006) 26:833–6. 16793614

[24] Martinez-Morillo M Lopez R Ibernon M Olive A. [Renal p-ANCA vasculitis in patients with systemic lupus erythematosus]. Med Clin. (2011) 137:379–80. 10.1016/j.medcli.2010.10.004 21145073

[25] Bertsias G Ioannidis JP Boletis J Bombardieri S Cervera R Dostal C. EULAR recommendations for the management of systemic lupus erythematosus. Report of a Task Force of the EULAR Standing Committee for International Clinical Studies Including Therapeutics. Ann Rheum Dis. (2008) 67:195–205. 10.1136/ard.2007.070367 17504841

